# Insulin and novel thioglycosides exert suppressive effect on human breast and colon carcinoma cells

**DOI:** 10.18632/oncotarget.23170

**Published:** 2017-12-11

**Authors:** Siddarth Agrawal, Marta Wozniak, Mateusz Luc, Kinga Walaszek, Ewa Pielka, Wieslaw Szeja, Gabriela Pastuch-Gawolek, Andrzej Gamian, Piotr Ziolkowski

**Affiliations:** ^1^ Department of Pathology, Wroclaw Medical University, Wroclaw, Poland; ^2^ Department of Organic Chemistry, Bioorganic Chemistry and Biotechnology, Silesian University of Technology, Gliwice, Poland; ^3^ Biotechnology Centre, Silesian University of Technology, Gliwice, Poland; ^4^ Department of Biochemistry, Wroclaw Medical University, Wroclaw, Poland

**Keywords:** cancer therapy, thioglycosides, insulin, breast cancer, colon cancer

## Abstract

The rationale for the implementation of novel therapies should be based on hallmarks of cancer. Two novel compounds labelled as thioglycoside A and B were designed and evaluated on breast and colon cancer cell lines. We assessed their cytotoxic effect after sensitizing cancer cells with insulin. In order to explore the underlying mechanisms, we performed tests to assess cell migration and motility, apoptosis, expression of glucose transporter 1 and proapoptotic proteins. Both compounds proved to have an antitumor effect which was significantly enhanced in combination with insulin. Linking glucose and anticancer agent presents an approach that exploits the Warburg effect. Targeting dysfunctional glycometabolism and increased glucose absorption is emerging as a promising anticancer strategy.

## INTRODUCTION

Malignancies of diverse origins deviate from healthy tissues in their high consumption of glucose. This phenomenon, recognized as one of the hallmarks of cancer, has attracted a great deal of interest in anticancer therapies. Conjugation of glucose with metabolic agents to selectively target cancer cells was inspired by the widespread use of radiolabeled glucose analog to visualize tumors and their metastases. The field of synthesis and evaluation of sugar-conjugated anticancer agents has grown significantly in recent years, with certain compounds in advanced clinical trials [1].

Thioglycosides have received considerable attention because they are widely employed as biological inhibitors [2–6], inducers [7–9]. Moreover, they are promising candidates in synthetic carbohydrate chemistry as convenient and versatile glycosyl donors. Among these, glycosyl donors are the thioglycosyl heterocycles that are sufficiently stable under a variety of reaction conditions and have the ability to be readily converted into a variety of other functionalities [10–12].

S-Glycosides are very attractive substitutes for O-glycosides, as it is well-known that they are much less susceptible to enzymatic cleavage as well as chemical degradation [13]. Also, they often exhibit a similar conformational solution and similar or even more potent bioactivities compared to the corresponding O-glycosides. (5-Nitro-2-pyridyl) 1-thioglycosides and obtained by their oxidation sulfoxides were assayed for cytotoxicity and *in vitro* antiviral properties against classical swine fever virus (CSFV). The best antiviral activity exhibited sulfoxide derivative of (5-nitro-2-pyridyl) 1-tiolactoside [14]. Also, glycoconjugates formed by the combination of (5-nitro-2-pyridyl) 1-thioglycosides and uridine derivatives showed significant activity against the Flaviviridae family [15]. Pyridine thioglycosides were reported as a new class of antimetabolites which exert inhibitory effects on both DNA and RNA containing viruses [16]. Thioglycosides have been proved to have good cytotoxic effects against Ehrlich ascites carcinoma cells (EAC cells) and four human cancer cell lines, namely liver Hepg2, breast MCF7, brain U251, lung H460. The postulated mechanism of action of pyridine thioglycosides is a cell cycle arrest in the S phase similar to the antimetabolites and cell cycle arrest in the G2/M phase (M phase) resembling microtubules inhibitors [17]. It was found that antitumour effectiveness of thioglycosides strongly depends on the structure of substituents in the pyridine ring [17]. On the other hand, result presented by Romero-Ramires et al confirmed the higher resistance to enzymatic hydrolysis of thioglycosides as compared to O-glycosyl derivatives. *in vivo* experiments in nude mice bearing an implanted C6 glioma showed that the thioglycoside reduced tumor volume, while the O-glycosyl derivative was inactive, highlighting the importance of using enzyme resistant glycosides [18]. Taking this into account in the planned study, negatively substituted 3-nitro and 5-nitro pirydyl thioglycosides resistance to hydrolysis were selected.

It is well established that insulin exhibits potent metabolic properties and is implicated in many malignancies [19]. Its impact on cellular uptake of many compounds including glucose by facilitated diffusion has been documented [20]. The use of insulin for cancer-specific treatment has been tested in several studies [21–25].

In this research, we have analyzed the antitumor effect of novel compounds: (5-nitro-2-pyridyl) 1-thio-β**-**D-glucopyranoside labelled as thioglycoside A, and (3-nitro-2-pyridyl) 1-thio-β**-**D-glucopyranoside labelled as thioglycoside B, on three cancer cell lines: MCF-7 human breast cancer cell line and human colon cancer cell lines: Caco-2, SW480.

We further assessed whether insulin can enhance the antitumor effect of these compounds. To investigate and establish the possible mechanisms of this phenomenon, we assessed cell proliferation, cell migration and motility, expression of glucose transporter 1 (GLUT-1) and proapoptotic proteins (caspase-3, BAX).

## RESULTS

### Thioglycoside A and B exhibit antitumor effect

**Figure 1:**
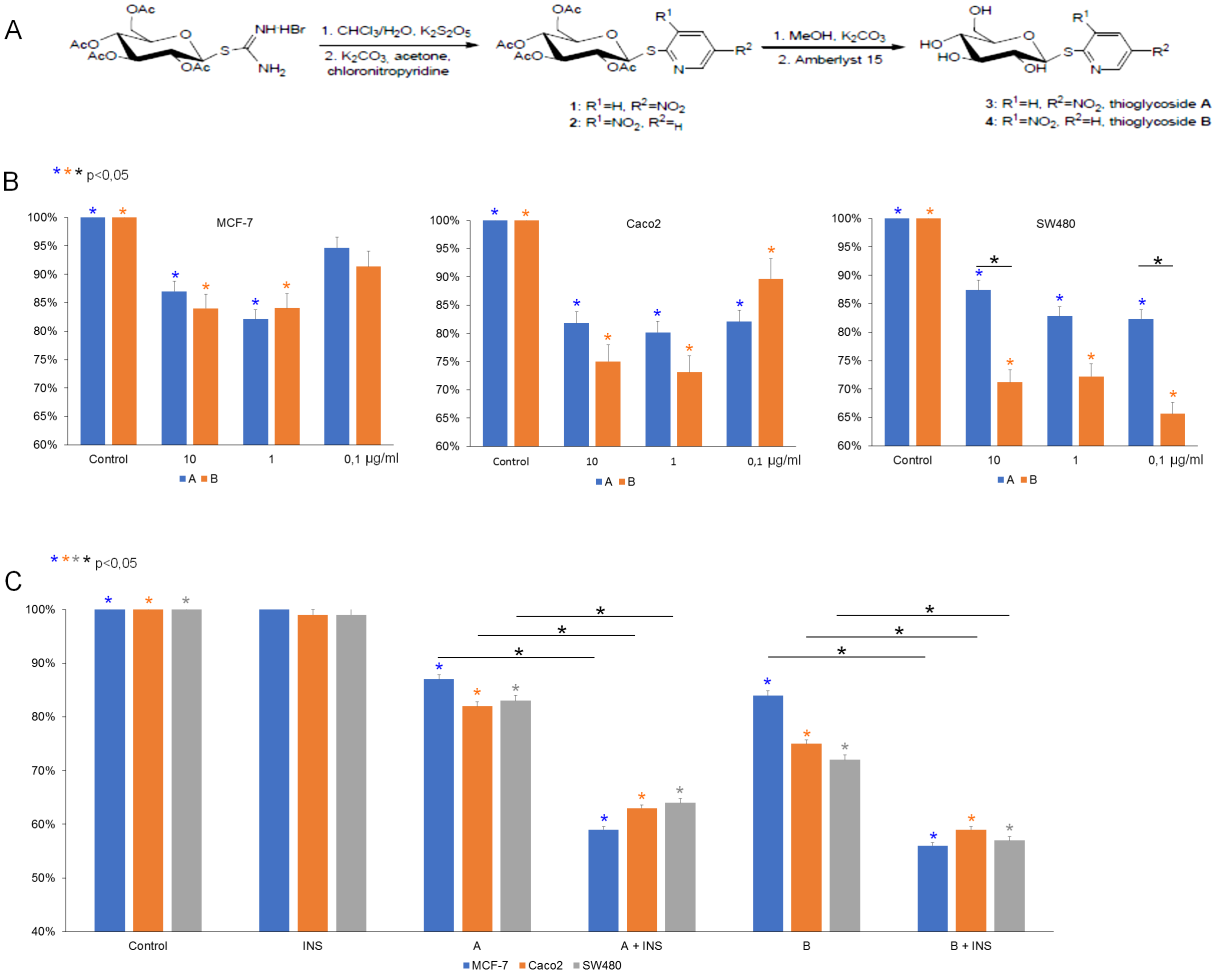
**(A)** Synthesis of compounds (5-nitro-2-pyridyl) 2,3,4,6-tetra-O-acetyl-1-thio-β-D-glucopyranoside (1, thioglycoside A) and (3-nitro-2-pyridyl) 2,3,4,6-tetra-O-acetyl-1-thio-β-D-glucopyranoside (2, thioglycoside B). **(B)** Activity of thioglycosides A and B on MCF-7, Caco-2, SW480 cancer cell lines. All three cell lines were treated with 10 μg/ml, 1 μg/ml, 0.1 μg/ml of thioglycoside A and B respectively for 24 hours. Cytotoxic effect was measured by MTT assay. Data are shown as mean ±SD from three separate experiments. **(C)** After 8-hour insulin pretreatment (40 μg/ml for MCF-7 and 100 μg/ml for Caco-2 and SW-480) all three cell lines were exposed to 10 μg/ml of thioglycosides A and B respectively for 24 hours. Inhibitory effect was measured by MTT assay. The results are shown as mean ±SD from three individual experiments. Statistically significant variables were marked with ^*^ (p<0,05).

To identify the optimal concentration of the compounds, various doses were tested. The thioglycosides in concentrations 10 μg/ml and 1 μg/ml exhibited significant inhibition in viability of breast and colon cancer cells (Figure 1B). The effect of both thioglycosides on MCF-7 and Caco-2 cell viability was similar. However, by statistical analysis we found that compound B is more cytotoxic to SW480 than compound A. The impact of non-conjugated glucose and other sugars on cell viability of breast cancer cells were assessed during preliminary studies. We found no significant changes in viability of the cells (Supplementary Material 1).

### Insulin enhances the inhibitory effect of thioglycosides

MCF-7 cancer cells were pretreated with 40 μg/ml insulin (INS), while colon cancer cells with 100 μg/ml. After incubation with insulin for 8 hours, cells were treated with thioglycosides A and B at concentration 10 μg/ml. Insulin alone had no significant effect on cell growth (Figure 1C). We found that the combination of INS and thioglycosides produced a significant inhibition in growth of both breast and colon cancer cells.

### Combination of insulin and thioglycosides inhibits cell motility

**Figure 2:**
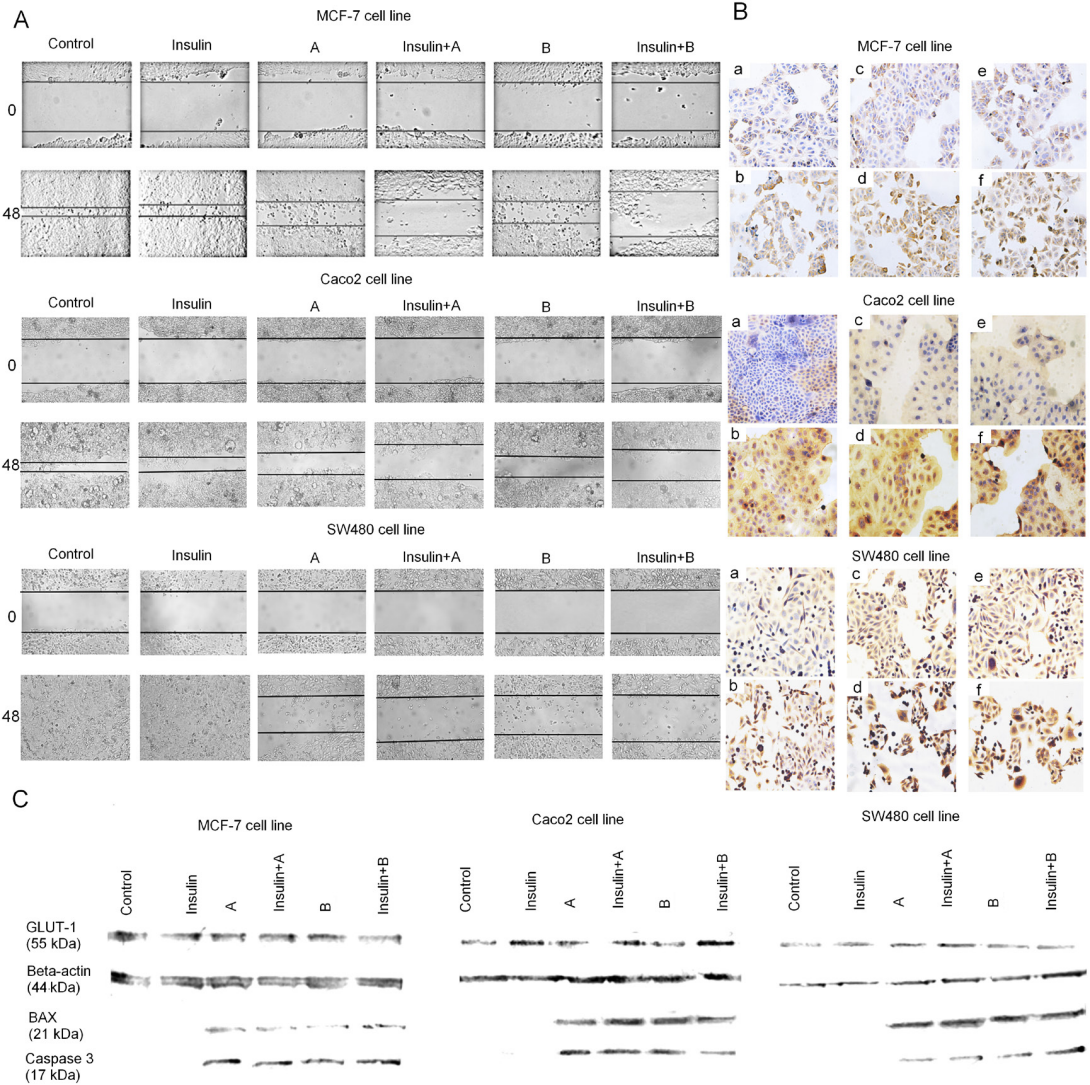
**(A)** Effect of thioglycosides A and B and thioglycosides A and B with additional insulin pretreatment on migration of MCF-7, Caco-2 and SW-480 cancer cells. Cell migration was evaluated through wound healing assay. Photomicrographs were taken at specific time points with an inverted microscope and digital camera. Control: MCF-7/Caco-2/SW480 cancer cells; Insulin: MCF-7/Caco-2/SW480 exposed to insulin (40 μg/ml for MCF-7 and 100 μg/ml for Caco-2 and SW-480); A: MCF-7/Caco-2/SW480 cancer cells treated with 10 μg/ml of thioglycoside A; A+Insulin: MCF-7 cancer cells pretreated with 40 μg/ml of insulin and Caco-2/SW480 with 100 μg/ml of insulin and treated with 10 μg/ml of thioglycoside A; B: MCF-7/Caco-2/SW480 cancer cells treated with 1 μg/ml of thioglycoside B; B+Insulin: MCF-7 cancer cells pretreated with 40 μg/ml of insulin and Caco-2/SW480 with 100 μg/ml of insulin and treated with 1 μg/ml of thioglycoside B. **(B)** Immunohistochemistry staining for GLUT-1 protein in MCF-7, Caco-2 and SW480 cancer cell lines. LSAB+ method, hematoxylin-counterstained at magnification 100x and 200x (a- control, b- INS, c- compound A, d- compound A+INS, e- compound B, f- compound B+INS). **(C)** Western blotting analysis of expression of apoptosis-related proteins and GLUT-1 receptor in MCF-7, Caco-2 and SW480 cancer cell lines. Cancer cells were incubated with insulin for 8 hours (40 μg/ml for MCF-7 and 100 μg/ml for Caco-2 and SW-480) and treated with 10 μg/ml of compound A and 1 μg/ml of compound B.

Wound-healing assay was performed to assess the combined effect of insulin and thioglycosides on cell proliferation and cell motility (Figure 2A). The results indicate that control and INS-treated cells almost completely filled the “wound” in MCF-7 and Caco-2 cells by 24h. The wound was filled completely in SW480 cells. In case of cells treated only with thioglycosides, a slightly hindered cell was observed. In sharp contrast, an addition of INS with thioglycosides significantly inhibited wound healing. This effect was observed in all cancer cell lines.

### Impact on GLUT-1 expression and apoptosis

The effect of insulin and compounds A and B on the expression of glucose transporter was analysed by immunocytochemistry. We found that treatment with insulin caused an elevated cytoplasmic expression of GLUT-1 protein when compared with the control. Comparing to the insulin alone, the combinations of thioglycosides with insulin, produced similar effects (Figure 2B). The findings were confirmed in Western blotting analysis (Figure 2C).

**Figure 3:**
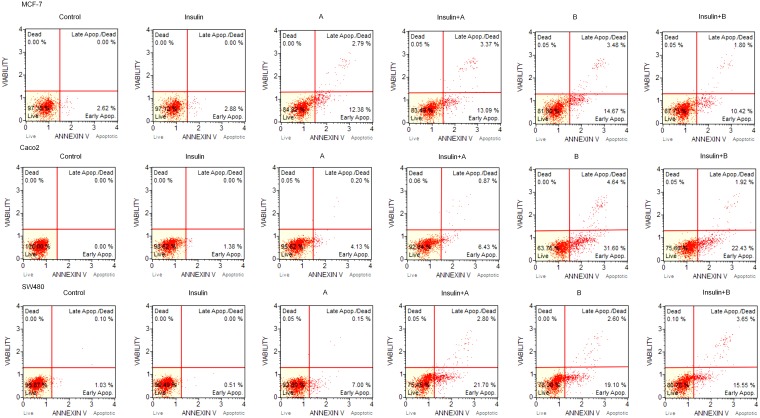
Original histogram plots include a percentage of live, early apoptotic, late apoptotic, total apoptotic, and dead cells differentiated using Muse^®^ Annexin V and Dead Cell Assay Kit

The expression of proapoptotic proteins – caspase 3 and BAX was analysed by Western blotting. We detected high levels of proapoptotic proteins in cells treated with combination of insulin and thioglycosides as well as thioglycosides only (Figure 2C). The flow cytometry analysis showed that insulin had no significant impact on the ratio of apoptosis in all tested cancer cell lines (Figure 3). Over 20% of the MCF-7 breast cancer cells underwent apoptosis when treated with compound A or B. The addition of insulin did not result in significant changes in the ratio of apoptotic cells. The level of apoptosis in SW480 cells treated with thioglycoside A was slightly over 10%. The addition of insulin to compound A resulted in an increased ratio of apoptotic cells, which was slightly over 26%. Compound B with and without insulin produced a similar effect (over 20% of cells undergoing apoptosis). The apoptotic action of compound A on colon cancer cell line Caco-2 was similar to that of SW480 (nearly 10%). The addition of insulin to compound A caused an enhancement of apoptosis by slightly over 6%. The highest level of apoptosis was detected in Caco-2 cells treated with compound B (nearly 34%). Interestingly, the combination of insulin and compound B produced 23,33% of apoptosis.

## DISCUSSION

Cancer cells, unlike the majority of somatic cells, consume large amounts of glucose and rely on aerobic glycolysis to generate ATP. This observation was described in the 1920s by Otto Warburg and is known as the Warburg effect [26, 27]. The phenomenon is a significant survival advantage as it allows the cancer cells to survive and multiply both in normoxic and hypoxic environment as well to evade immune killing [28, 29].

Glucose as a main fuel for ATP production needs to be transported from extracellular space via cell membrane. Diffusion of extracellular glucose is facilitated by membrane proteins called glucose transporters (GLUTs). Most of the cells which underwent malignant transformation present overexpression of GLUT family members, especially GLUT-1 [30]. Targeting dysfunctional glycometabolism and marked glucose absorption is emerging as a promising anticancer strategy [28]. Linking glucose and anticancer agent presents an approach that exploits the Warburg effect.

Insulin is known to be implicated in many malignancies [19]. It affects the cell metabolism by increasing transcription, stimulating DNA synthesis as well as increasing the turnover of cellular carbohydrates and lipids [20]. The cellular uptake of glucose and numerous ions is markedly enhanced in the presence of insulin. Due to its complex influence on malignant cell metabolism, insulin has been exploited as a potential sensitizing agent in cancer therapy. Several *in vitro* and clinical studies have found that the inhibitory effect of various cytotoxic agents can be enhanced in the presence of insulin [21–25].

Herein we report the discovery of novel compounds in which sugar is linked to aglycone in an efficient manner through S-glycosidic bond. Moreover, this is the first study that investigated the combined use of insulin and glucose linked to anticancer agent. Our research conducted on breast and colon cancer cell lines demonstrated cytotoxic activity of thioglycoside A and thioglycoside B. Interestingly, this effect was significantly enhanced in the presence of insulin. We found that insulin can increase the cytotoxic action of glucose-conjugates up to two-fold. These potential candidates for future anticancer therapy displayed antiproliferative as well as proapoptotic action *in vitro*. These results can be explained by: 1/ inhibition of metabolic pathways that lead to formation of purines and pyrimidines as well as 2/ inhibition of tubulin polymerization [17].

In present study, we assessed the impact of insulin on GLUT-1 expression. The results showed an elevated expression of GLUT-1 in insulin treated cells. These findings are consistent with previous studies [25]. We suggest that an overexpression of GLUTs is responsible for a higher uptake of the novel compounds, thus leading to enhanced cytotoxicity. It is hypothesized that the preferential uptake of glucose into malignant versus normal tissues, which is further enhanced by insulin, is responsible for the observed anticancer effect.

In conclusion, our study demonstrates the pioneer use of novel thioglycosides with supplementary insulin to selectively target cancer cells *in vitro*. Further *in vivo* studies are required to assess its application. This approach has a great deal of potential and a considerable opportunity for growth.

## MATERIALS AND METHODS

### Chemistry

The synthesis and assessment of antitumor activity of number of thioglycoside derivatives of substituted dihydropyridine have been performed. From the results of studies on the antitumor activity and structure-activity relationship, it can be concluded that the effect of the substituent in the aryl group placed at position 4 as well as the glycopyranosylthio moiety in the pyridine ring was obvious [31]. Many methods are presented for the efficient preparation of thioglycosides [32]. The conventional synthesis of dihydropyridine thioglycosides is achieved through the reaction of piperidinium salts of dihydropyridine thiolates with per-*O*-acetyl glycopyranosyl halides. Treatment of the glycosides with saturated solution of ammonia in methanol at room temperature afforded the free glycosides. A simple approach for the stereoselective synthesis of 1,2-trans 1-thioglycosides is based on the utilization of glycosyl isothiourea derivatives as precursors. Conversion of glycosyl isothiourea into per-*O*-acetyl-1-thio-β-D-hexopyranoses followed by treatment with substituted aryl chlorides under basic conditions provides an efficient method for the synthesis of aryl 1-thio-β-glycosides [33]. We have found that this method is an effective procedure of the synthesis of hetaryl thioglycosides (Figure 1A).

### General information

The ^1^H NMR and ^13^C NMR spectra were recorded with an Agilent spectrometer at a frequency of 400 MHz using TMS as an internal standard and CDCl_3_ or CD_3_OD as solvents. NMR solvents were purchased from ACROS Organics (Geel, Belgium). Chemical shifts (δ) are expressed in ppm and coupling constants (*J*) in Hz. The NMR spectra are shown in the Supplementary Material 2. Optical rotations were measured with a JASCO P-2000 polarimeter using a sodium lamp (589.3 nm) at room temperature. Melting point measurements were performed on OptiMelt (MPA 100) Stanford Research Systems. Mass spectra were recorded with a WATERS LCT Premier XE system (high resolution mass spectrometer with TOF analyzer) or with a 4000 QTRAP ABSciex mass spectrometer using electrospray-ionization (ESI) technique. Reactions were monitored by TLC on precoated plates of silica gel 60 F254 (Merck Millipore). The TLC plates were inspected under UV light (λ = 254 nm) or charring after spraying with 10% sulfuric acid in ethanol. Crude products were purified using column chromatography performed on Silica Gel 60 (70-230 mesh, Fluka) developed with toluene/EtOAc or CHCl_3_/MeOH solvent systems. All evaporations were performed on a rotary evaporator under diminished pressure at 40 °C.

Isothiouronium salt of tetra-*O*-acetyl-β-D-glucose was prepared as described in the literature [34]. All chemicals used in experiments were of analytical grade and purchased from Sigma-Aldrich, Fluka and ACROS Organics.

#### General procedure for synthesis of per-*O*-acetylated nitropyridyl 1-thioglycosides

Synthesis of (5-nitro-2-pyridyl) and (3-nitro-2-pyridyl) per-*O*-acetyl-1-thio-β-D-glucosides (**1)** [35] and (**2**) [36], respectively, was performed from the isothiouronium salt of tetra-*O*-acetyl-β-D-glucose according to the following procedure.

A solution of K_2_S_2_O_5_ (6.84 g, 82 mmol) in water (60 mL) was heated up to 80°C for 2 minutes. After cooling down to 50°C, the isothiouronium salt of tetra-*O*-acetyl-β-D-glucose (15 g, 30.8 mmol) in CHCl_3_ (200 mL) was added to this solution. The resulting mixture was heated at the reflux for 0.5 h. Then the layers were separated, the organic layer was washed with brine (3×100 mL), dried over anhydrous MgSO_4_ and concentrated *in vacuo*. The residue was dissolved in dry acetone (500 mL). To this solution were added sequentially 2-chloro-5-nitropyridine or 2-chloro-3-nitropyridine (4.87 g, 31 mmol) and K_2_CO_3_ (8.28 g, 60 mmol). The resulting mixture was stirred at room temperature for 0.5 or 2 h, then filtered and evaporated. The crude products were purified by crystallization from ethyl alcohol. Residue after crystallization was purified by column chromatography (toluen: ethyl acetate; gradient: 10:1 to 4:1).

#### (5-nitro-2-pyridyl) 2,3,4,6-tetra-*O*-acetyl-1-thio-β-D-glucopyranoside (1, thioglycoside A)

Reaction time: 30 minutes. Product **1** (12.3 g, 82%) was obtained as a white solid after purification by a crystallization from ethyl alcohol. ${\left[\alpha \right]}_{n}^{20}$ = 14 (CHCl_3_, c = 5); m.p. 181-183°C; ^1^H NMR (400 MHz, CD_3_OD): δ 2.02, 2.03, 2.04, 2.05 (4s, 12H, CH_3_CO), 3.92 (ddd, 1H, *J* = 2.4 Hz, *J* = 4.7 Hz, *J* = 10.2 Hz, H-5), 4.11 (dd, 1H, *J* = 2.4 Hz, *J* = 12.5 Hz, H-6a), 4.27 (dd, 1H, *J* = 4.7 Hz, *J* = 12.5 Hz, H-6b), 5.17 (dd, 1H, *J* = 9.0 Hz, *J* = 10.2 Hz, H-4), 5.25 (dd, 1H, *J* = 9.8 Hz, *J* = 10.6 Hz, H-2), 5.38 (dd, 1H, *J* = 9.0 Hz, *J* = 9.8 Hz, H-3), 6.11 (d, 1H, *J* = 10.6 Hz, H-1), 7.34 (d, 1H, *J* = 8.8 Hz, H-3_pyr_), 8.31 (dd, 1H, *J* = 2.6 Hz, *J* = 8.8 Hz, H-4_pyr_), 9.27 (d, 1H, *J* = 2.6 Hz, H-6_pyr_).^13^C NMR (100 MHz, CD_3_OD): δ 20.08, 20.10, 20.19 (CH_3_CO), 61.29 (C-6), 67.56, 68.62, 73.42, 75.72 (C-2, C-3, C-4, C-5), 80.55 (C-1), 121.82, 130.68, 141.56, 144.52, 163.09 (C_pyr_), 168.89, 168.94, 169.57, 170.02 (CO).

#### (3-nitro-2-pyridyl) 2,3,4,6-tetra-*O*-acetyl-1-thio-β-D-glucopyranoside (2, thioglycoside B)

Reaction time: 2 hours. Product **2** (12.7 g, 85%) was obtained as a white solid after purification by a crystallization from ethyl alcohol. ${\left[\alpha \right]}_{n}^{20}$ = 64 (CHCl_3_, c = 1); m.p. 125-126°C; ^1^H NMR (400 MHz, CD_3_OD): δ 2.02, 2.03, 2.04, 2.05 (4s, 12H, CH_3_CO), 3.92 (ddd, 1H, *J* = 2.4 Hz, *J* = 4.7 Hz, *J* = 10.2 Hz, H-5), 4.23 (dd, 1H, *J* = 2.4 Hz, *J* = 12.5 Hz, H-6a), 4.23 (dd, 1H, *J* = 4.7 Hz, *J* = 12.5 Hz, H-6b), 5.18 (dd, 1H, *J* = 9.0 Hz, *J* = 10.2 Hz, H-4), 5.32 (dd, 1H, *J* = 9.4 Hz, *J* = 10.2 Hz, H-2), 5.38 (dd, 1H, *J* = 9.0 Hz, *J* = 9.4 Hz, H-3), 6.07 (d, 1H, *J* = 10.2 Hz, H-1), 7.32 (dd, 1H, *J* = 4.7 Hz, *J* = 8.2 Hz, H-5_pyr_), 8.53 (dd, 1H, *J* = 1.6 Hz, *J* = 8.2 Hz, H-6_pyr_), 8.73 (dd, 1H, *J* = 1.6 Hz, *J* = 4.7 Hz, H-4_pyr_).^13^C NMR (100 MHz, CD_3_OD):δ 20.26, 20.59, 20.66 (CH_3_CO), 61.85 (C-6), 68.20, 68.90, 74.38, 75.96 (C-2, C-3, C-4, C-5), 79.85 (C-1), 120.09, 133.95, 142.32, 152.98, 154.33 (C_pyr_), 169.23, 169.34, 170.22, 170.56 (CO).

#### Deprotection of (5-nitro-2-pyridyl) or (3-nitro-2-pyridyl) per-*O*-acetylated 1-thioglycosides

Compounds **1** or **2** (5 g, 10.2 mmol) were suspended in MeOH (300 mL). To the resulting mixture K_2_CO_3_ (5.5 g, 40 mmol) was added. The whole mixture was stirred at room temperature. The reaction was monitored by TLC on silica gel plates using CHCl_3_: MeOH (5:1, v/v) solvent system. After the completion of the reaction, the solid was filtered off, washed with MeOH and filtrate was neutralized by adding ion exchange resin Amberlyst 15. The resin was filtered off and the organic layers were concentrated. For purification of compound **4** the residue was redissolved in a MeOH (10 mL) and concentrated with a small amount of silica-gel in order to prepare sample for purification by column chromatography.

#### (5-nitro-2-pyridyl) 1-thio-β-D-glucopyranoside (3)

Reaction time: 30 minutes. Product **3** (4.79 g, 95%) was obtained as a white solid after purification by a crystallization from anhydrous ethanol. ${\left[\alpha \right]}_{n}^{20}$ = 110 (MeOH, c = 0.8); m.p. 60-64°C; ^1^H NMR (400 MHz, CD_3_OD): δ 3.43-3.51 (m, 2H, H-2, H-4), 3.54 (ddd, 1H, *J* = 2.2 Hz, *J* = 5.8 Hz, *J* = 9.3 Hz, H-5), 3.55 (dd, 1H, *J* = 8.6 Hz, *J* = 9.4 Hz, H-3), 3.73 (dd, 1H, *J* = 5.8 Hz, *J* = 12.1 Hz, H-6a), 3.92 (dd, 1H, *J* = 2.2 Hz, *J* = 12.1 Hz, H-6b), 5.51 (d, 1H, *J* = 9.8 Hz, H-1), 7.63 (dd, 1H, *J* = 0.8 Hz, *J* = 9.0 Hz, H-3_pyr_), 8.46 (dd, 1H, *J* = 2.7 Hz, *J* = 9.0 Hz, H-4_pyr_), 9.26 (d, 1H, *J* = 2.7 Hz, H-6_pyr_).^13^C NMR (100 MHz, CD_3_OD):δ 62.87. (C-6), 71.43, 73.77, 79.97, 82.49 (C-2, C-3, C-4, C-5), 85.41 (C-1), 123.31, 132.74, 143.36, 145.89, 167.90 (C_pyr_).

#### (3-nitro-2-pyridyl) 1-thio-β-D-glucopyranoside (4)

Reaction time: 30 minutes. Product **4** (4.94 g, 98%) was obtained as a solidifying oil after purification by a column chromatography (chloroform: methanol; gradient: 100:1 to 10:1). ${\left[\alpha \right]}_{n}^{20}$ = 59 (MeOH, c = 0.8); ^1^H NMR (400 MHz, CD_3_OD): δ 3.36-3.45 (m, 2H, H-4, H-5), 3.45-3.52 (m, 2H, H-2, H-3), 3.63 (dd, 1H, *J* = 5.1 Hz, *J* = 12.1 Hz, H-6a), 3.86 (dd, 1H, *J* = 1.9 Hz, *J* = 12.1 Hz, H-6b), 5.81 (m, 1H, H-1), 7.37 (dd, 1H, *J* = 4.7 Hz, *J* = 8.2 Hz, H-5_pyr_), 8.55 (dd, 1H, *J* = 1.6 Hz, *J* = 8.2 Hz, H-6_pyr_), 8.76 (dd, 1H, *J* = 1.6 Hz, *J* = 4.7 Hz, H-4_pyr_).^13^C NMR (100 MHz, CD_3_OD): δ 62.67 (C-6), 71.34, 73.21, 80.14, 82.17 (C-2, C-3, C-4, C-5), 83.77 (C-1), 121.20, 134.97, 144.01, 154.56, 156.30 (C_pyr_).

### *In vitro* analysis of biological activity

#### Cell culture and experiment conditions

The human breast cancer cell line MCF-7, the human colon cancer cell lines Caco-2 and SW480 were obtained from Leibniz Institute DSMZ-German Collection of Microorganisms and Cell Cultures. MCF-7 and SW480 cell lines were cultured in DMEM/F12 + heat-inactivated 10% foetal bovine serum + 1% glutamine and Caco-2 was cultured in 80% MEM (with Earle's salts) + 20% heat inactivated foetal bovine serum + 1% glutamine. Cells were incubated at 37°C in a 5% CO_2_ and a 95% humidified atmosphere. When cells reached 80% confluence, they were digested with 0.25% trypsin for the following experiments. Cell culture reagents were obtained from Gibco, Invitrogen (Thermo Fisher Scientific Inc., Carlsbad, CA, USA). For all of the experiments, the cells were cultured 24 hours after seeding for adherence and the culture medium was replaced. The next day, the cells were exposed to insulin (Insulin solution human, Sigma Aldrich, Germany) for 8 hours in dose 40 μg/ml for MCF-7 and 100 μg/ml for Caco-2 and SW480 and then treated for a further 24 hours by synthesized compounds in an optimal concentration evaluated after the viability assay. Test solutions of the tested compounds (1 mg/ml) were prepared by dissolving the substances in 100 μl of the dimethyl sulfoxide (DMSO, BioShop Canada Inc., Ontario, Canada) complemented with 900 μl of the tissue culture medium. Afterwards, the tested compounds were diluted in the culture medium to reach the final concentrations. The synthesized compounds 1 and 2 were further used in studies of their biological activities as compounds A and B, respectively.

#### Cell viability and proliferation assay

The viability of MCF-7, Caco-2, SW480 cells in response to thioglycoside A and B was determined by the 3-(4,5-dimethylthiazol-2-yl)-2,5-diphenyltetrazolium bromide (MTT) reduction assay. To assess the proper drug concentration, cells were seeded at a density of 7x10^3^/well in 96-well culture plates and treated as described in experimental conditions with different thioglycosides concentrations 10 μg/ml, 1 μg/ml, 0,1 μg/ml for 24 hours. MTT solution (Sigma Aldrich, Germany) was added to the wells on a 96-well plate to a final concentration of 0.5mg/ml and incubated at 37°C for 4h. Following incubation, the formazan crystals were solubilized with 100 μl DMSO (Sigma Aldrich, Germany) for 15 minutes. The optical absorbance (A) was measured at 490nm using a BioTek ELX800 multi-well reader (BioTek, Winooski, VT, USA). The absorbance in the untreated control group was regarded as 100% cell viability. The percentage of viable cells (VC) was calculated according to: VC (%) = (A of experimental group/A of control group)x100. All assays were repeated 3 times. For further experiments a concentration of 10 μg/ml and 1 μg/ml of compound A and B respectively, was used.

#### Immunocytochemistry

For the immunocytochemistry analysis, cells were seeded at 4 × 10^4^ cells per well in 4-well imaging slides (Eppendorf, Hamburg, Germany). After treatment, cells were placed in 4% paraformaldehyde at 4°C for 10 min, washed with PBS and permeabilized in 0.1% Tween 20 in PBS for 10 min. Immunocytochemistry was performed using the LSAB+ method (LSAB+ System HRP from DAKO, Glostrup, Denmark). After permeabilization, the cells were washed with PBS and incubated with the endogenous peroxidase-blocking buffer and then were incubated with the protein-blocking buffer. Next, the primary antibody against GLUT-1 receptor (Atlas Antibodies, Stockholm Sweden, dilution 1:100) was used, and slides were stored overnight at 4°C. The following day, the slides were washed with PBS and incubated for 1 hour with a secondary anti-rabbit-HRP conjugated antibody from DAKO Kit. Then the slides were rinsed twice with PBS and stained with 3,3’-diaminobenzidine in chromogen solution. Finally, the cells were counterstained with Mayer’s haematoxylin and then dehydrated in graded alcohols, cleared in xylene, and mounted with xylene-based mounting medium. The negative control was obtained by omitting the first antibody. Images of immunocytochemistry results were taken by a light microscope fitted with a digital camera (Nikon Eclipse 80i with camera DS-Fil-U2, Amsterdam, The Netherlands) at magnification of 100x and 200x.

### Western blotting analysis

The cells were plated at a density of 8x10^4^/well in 6-well culture plates. The cells were washed twice with pre-cooled PBS and, subsequently, cell lysates were prepared using RIPA buffer containing protease and phosphatase inhibitors (1% cocktails, all from Sigma Aldrich, Germany). The lysates were incubated with low agitation for 30 min at 4°C and then cleaned by centrifugation at 16 000xg for 15 minutes. The supernatants were collected, and the protein concentration was measured at 280nm using the Qubit Protein Assay for the Qubit Fluorometer (Invitrogen, Thermo Fisher Scientific Inc., Carlsbad, CA, USA).

Total 50 ug protein extracts were separated on 4% to 12% SDS-PAGE (sodium dodecyl sulfate polyacrylamide gel electrophoresis, all Western blotting reagents and equipment from Invitrogen, Thermo Fisher Scientific Inc., Carlsbad, CA, USA) and transferred to the nitrocellulose membrane. The membrane was blocked with phosphate-buffered saline containing 0.1% Tween 20 (Sigma Aldrich, Germany) with 10% bovine serum albumin (Sigma Aldrich, Germany) for 1h at room temperature. Subsequently, the membrane was incubated overnight at 4°C with the first antibodies’ solution. The primary antibodies used in this study included anti-β-actin for protein normalization (dilution 1:1000, Abcam, Cambridge, UK), anti-caspase 3 (dilution 1:500, Abcam, Cambridge, UK), anti-bax (dilution 1:1000, Abcam, Cambridge, UK), anti-GLUT1 (dilution 1:500, Atlas Antibodies, Stockholm, Sweden). After washing twice with PBS, the membrane was incubated with horseradish peroxidase-labelled secondary anti-rabbit antibody in dilution 1:1000 (Santa Cruz Biotechnology, Inc., Santa Cruz, CA, USA) for 1h at room temperature and thereafter washed three times with PBS. The final detection was performed with enhanced colorimetric Western blotting visualization reagent using the 1-Step Ultra TMB Blotting Solution (Thermo Fisher Scientific Inc., Carlsbad, CA, USA). The results were documented using appropriate Bio-Rad equipment (Molecular Imager Gel Doc TMXR+, BioRad, Hercules, CA, USA).

### Wound-healing assay

The cells were seeded in the 2 Well Culture Insert in u-Dish (Ibidi, Martinsried, Germany) according to manufacturer’s instructions. After appropriate cell attachment (24 hours), Culture Inserts were gently removed and the cells were treated either with cell medium or were exposed to 10 μg/ml or 1 μg/ml of compound A and B, with or without 8 h of insulin sensitization (40 μg/ml for MCF-7, and 100 μg/ml for Caco-2, SW480). Images of cell migration in 500 μm gap were taken at time point 0 hours and over a 24-hours time period by phase-contrast microscope fitted with a digital camera (Nikon Eclipse 80i with camera DS-Fil-U2, Amsterdam, The Netherlands) at magnification of 40x.

### Flow cytometry analysis

The ratio of apoptosis was measured using Muse^®^ Annexin V and Dead Cell Assay Kit. The cells were detached from their culture vessel (each well of 24-well plate), using Gibco™ Trypsin-EDTA as a dissociation reagent. The amount of 100 μl of Gibco™ Trypsin-EDTA was added to each well. After 5 minutes of incubation in 37°C, the cells were gently scraped off and the amount of 300 μl of medium (appropriate for each cell line) was added. The dissociated cells were transferred to Eppendorf^®^ tubes of 1.5 ml volume and gently mixed on vortex. Then the amount of 100 μL of each cell sample was added to 100 μl of Muse™ Annexin V & Dead Cell reagent and gently mixed. After 20 minutes of incubation at room temperature in darkness, samples were gently mixed and then loaded onto the Muse™ Cell Analyzer, where the test of degree of apoptosis was performed following the Muse™ Annexin V & Dead Cell Kit User’s Guide. The obtained results include a percentage of live, early apoptotic, late apoptotic, total apoptotic, and dead cells.

### Statistical analysis

The conformity of distribution of analyzed parameters with the normal distribution was checked. The conformity was assessed by the Shapiro–Wilk test. The homogeneity of variance was tested with Bartlett’s test. The significance of differences in mean values (M) in more than two populations for parameters of normal distribution and homogeneous variances was assessed with analysis of variance (ANOVA). In case of rejection of the null hypothesis of homogeneity of variance, to verify the differences between the mean values in pairs, Scheffe’s test was performed. The critical significance level was set at p=0.05. The data were expressed as mean ± standard deviation (M ± SD) and analyzed with the statistical program STATISTICA v.12 (StatSoft, Inc., Tulsa, OK, USA).

## SUPPLEMENTARY MATERIALS FIGURES


