# Supplementary material for: Insulin and novel thioglycosides exert suppressive effect on human breast and colon carcinoma cells
Source: Oncotarget. 2017 Dec 11;8(69):114173–82. doi: 10.18632/oncotarget.23170 (PMC5768394; doi:10.18632/oncotarget.23170)
Supplement: Supplementary file 1 [file oncotarget-08-114173-s001.pdf]

Insulin and novel thioglycosides exert suppressive effect on human breast and colon carcinoma cells

SUPPLEMENTARY MATERIALS

Supplementary Material 1

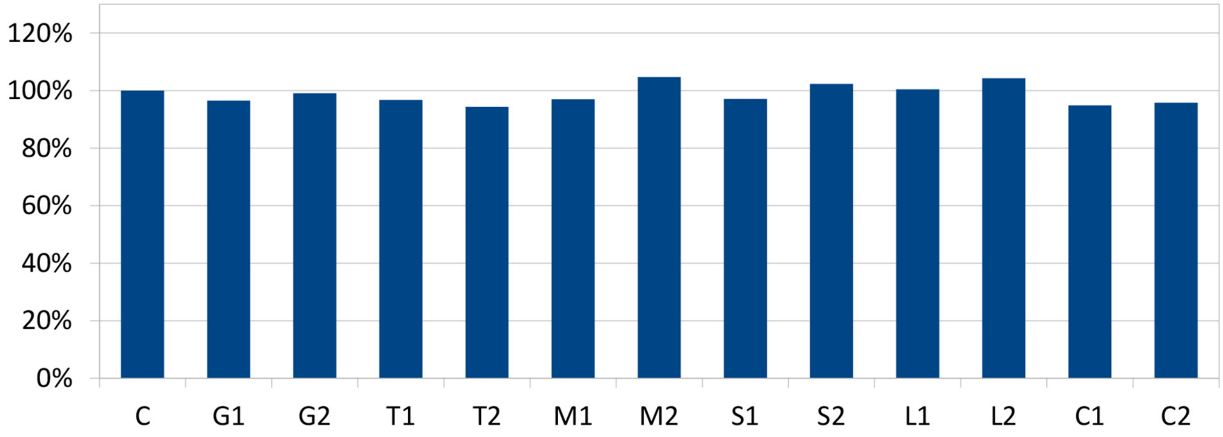

The impact of various sugars on cell viability of MCF-7 breast cancer cell line. Cells were treated for 24 hours with sugars in two different concentrations: glucose: 1 - 0.556 mM and 2 - 0.056 mM; trehalose, maltose, saccharose, lactose and cellobiose: 1 - 0.292 mM and 2 - 0.029 mM.
C - control; G - glucose; T - trehalose; M - maltose; S - saccharose; L - lactose; C - cellobiose. No statistical significance was found in viability of the cells.

Supplementary Material 2

^{1}H and ^{13}C NMR spectra of the analyzed compounds.

1. Spectra

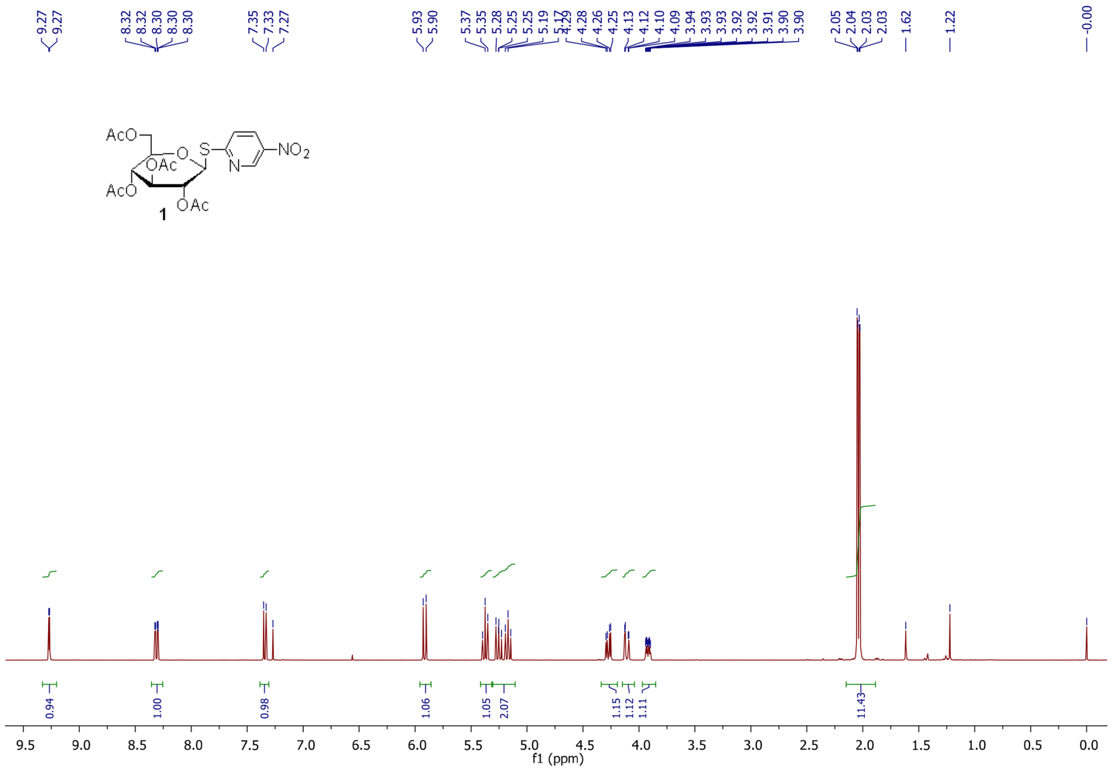

Supplementary Figure 1: ^{1}H NMR spectrum of (5-nitro-2-pyridyl) 2,3,4,6-tetra-*O*-acetyl-1-thio-β-D-glucopyranoside 1.

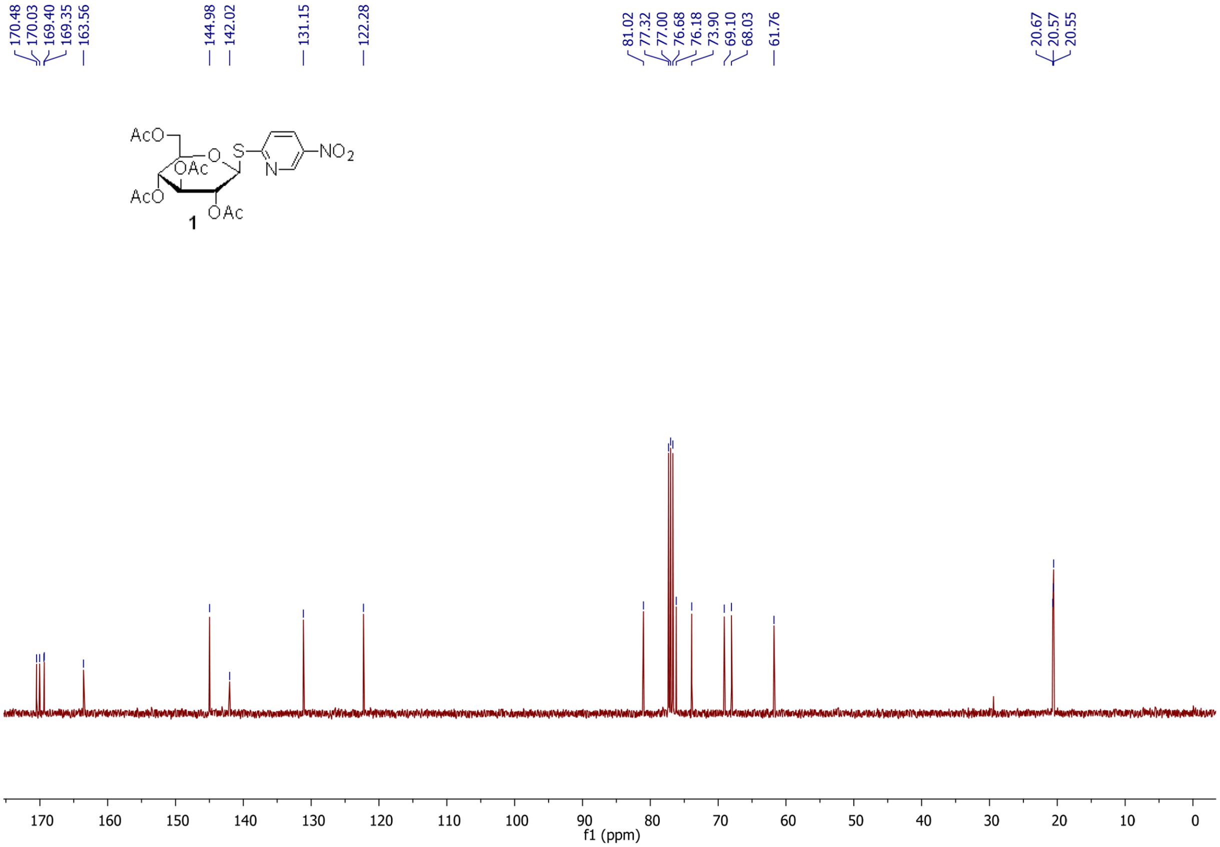

Supplementary Figure 2: ^{13}C NMR spectrum of (5-nitro-2-pyridyl) 2,3,4,6-tetra-*O*-acetyl-1-thio-β-D-glucopyranoside 1.

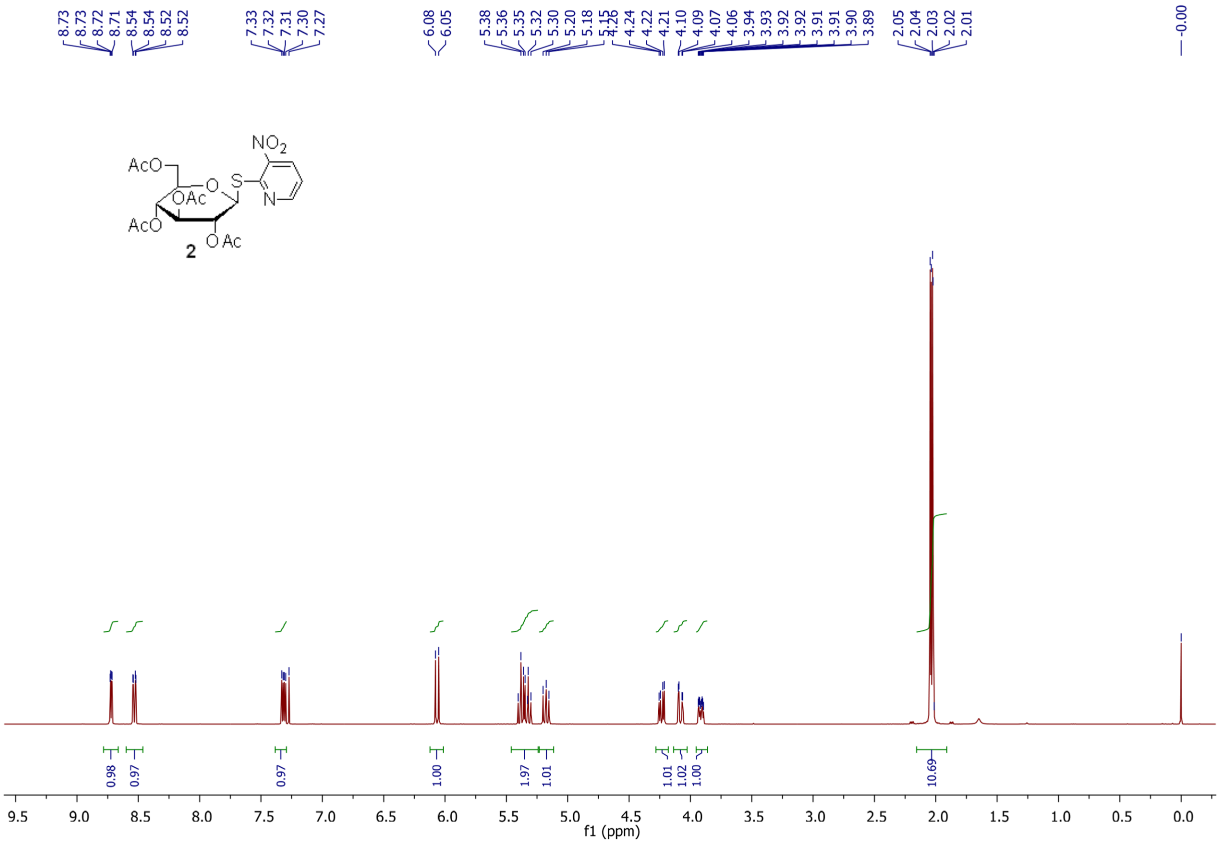

Supplementary Figure 3: ^{1}H NMR spectrum of (3-nitro-2-pyridyl) 2,3,4,6-tetra-*O*-acetyl-1-thio-β-D-glucopyranoside 2.

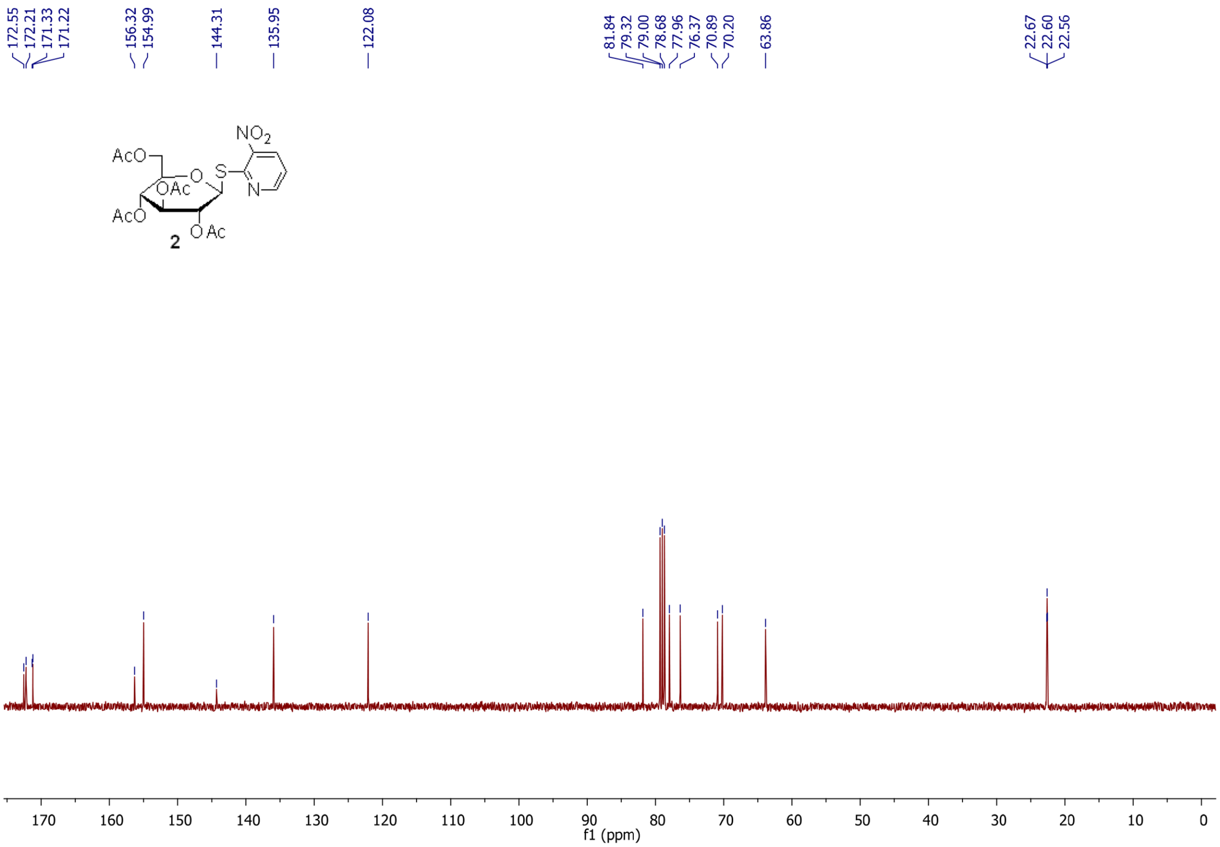

Supplementary Figure 4: ^{13}C NMR spectrum of (3-nitro-2-pyridyl) 2,3,4,6-tetra-*O*-acetyl-1-thio-β-D-glucopyranoside 2.

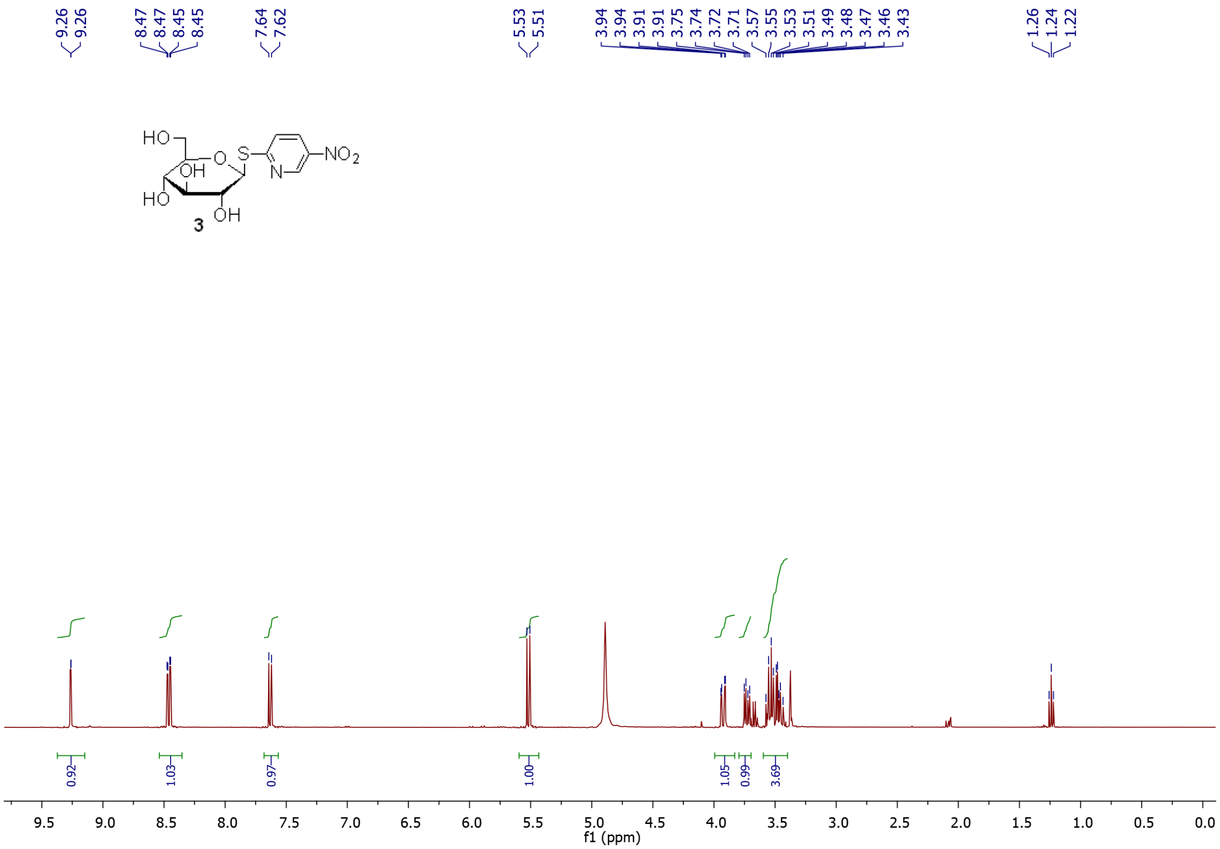

Supplementary Figure 5: ^{1}H NMR spectrum of (5-nitro-2-pyridyl) 1-thio-β-D-glucopyranoside 3.

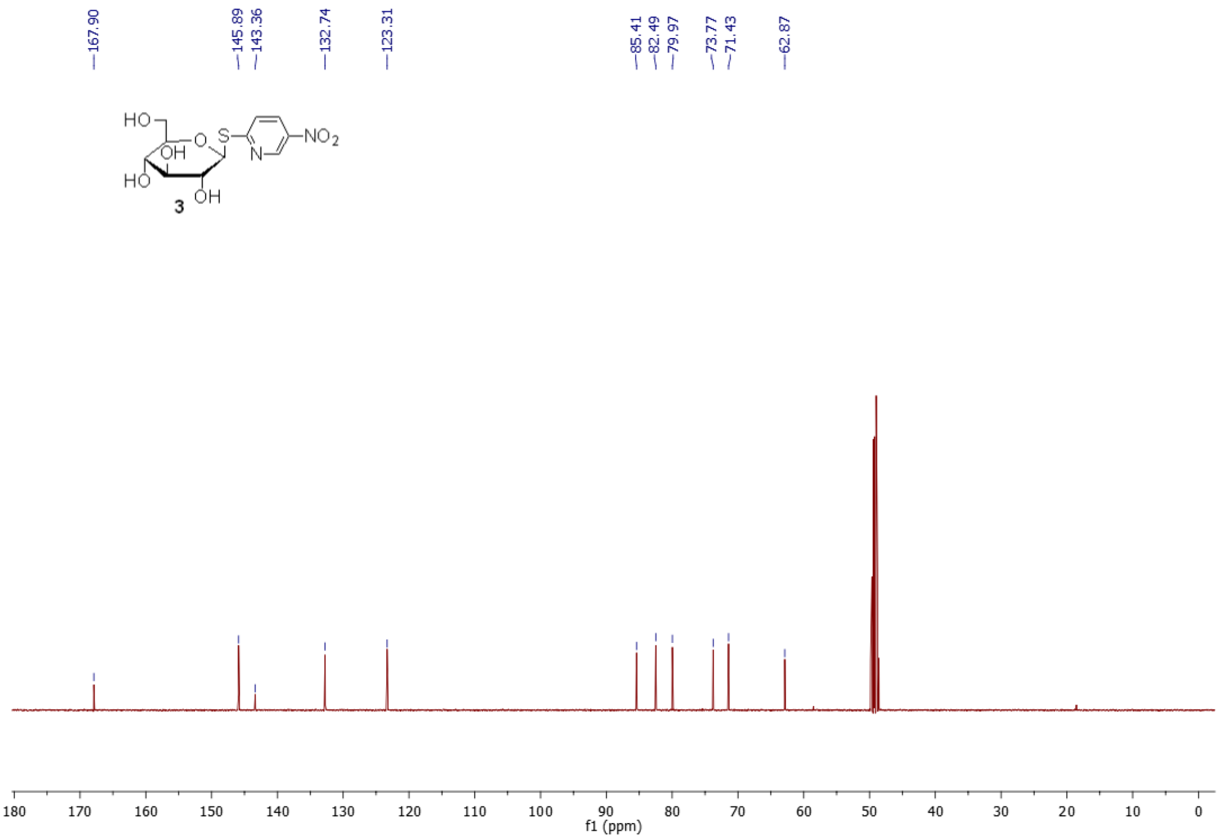

Supplementary Figure 6: ^{13}C NMR spectrum of (5-nitro-2-pyridyl) 1-thio-β-D-glucopyranoside 3.

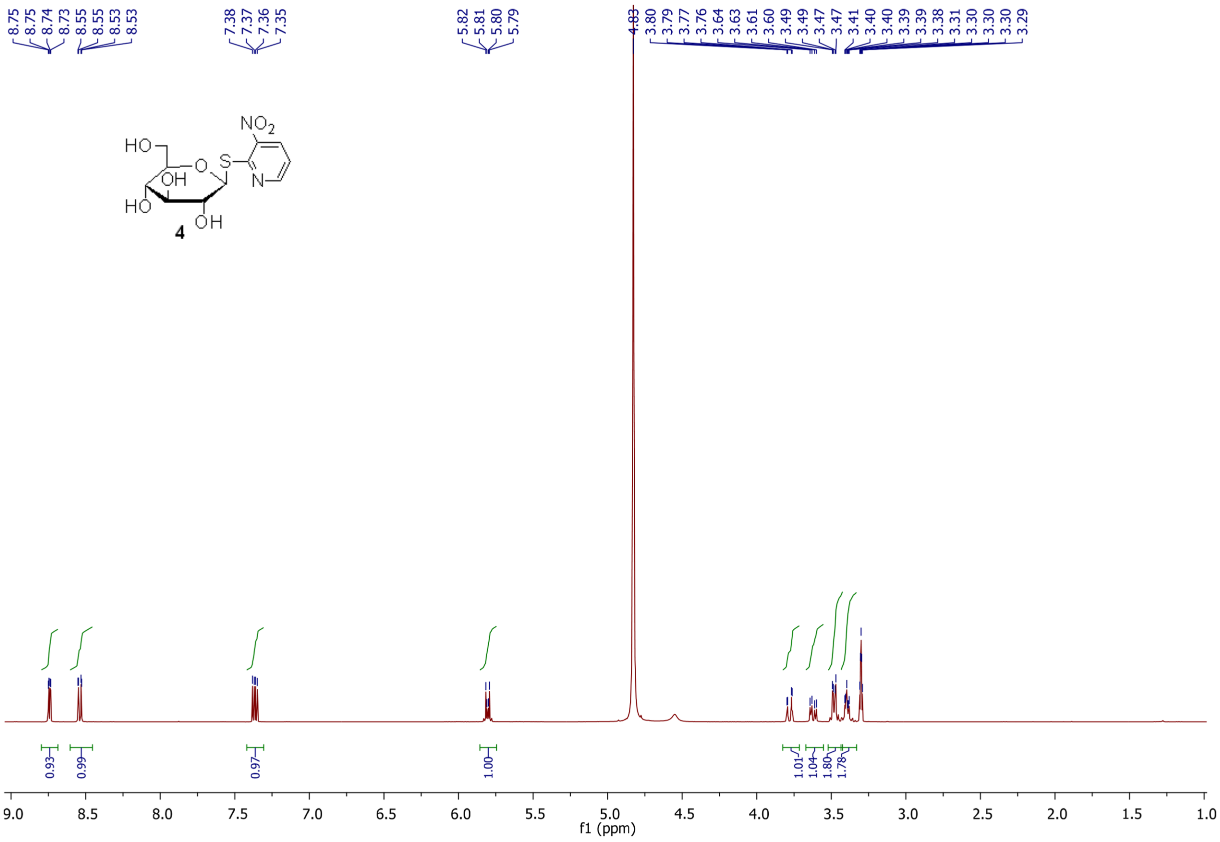

Supplementary Figure 7: ^{1}H NMR spectrum of (3-nitro-2-pyridyl) 1-thio-β-D-glucopyranoside 4.

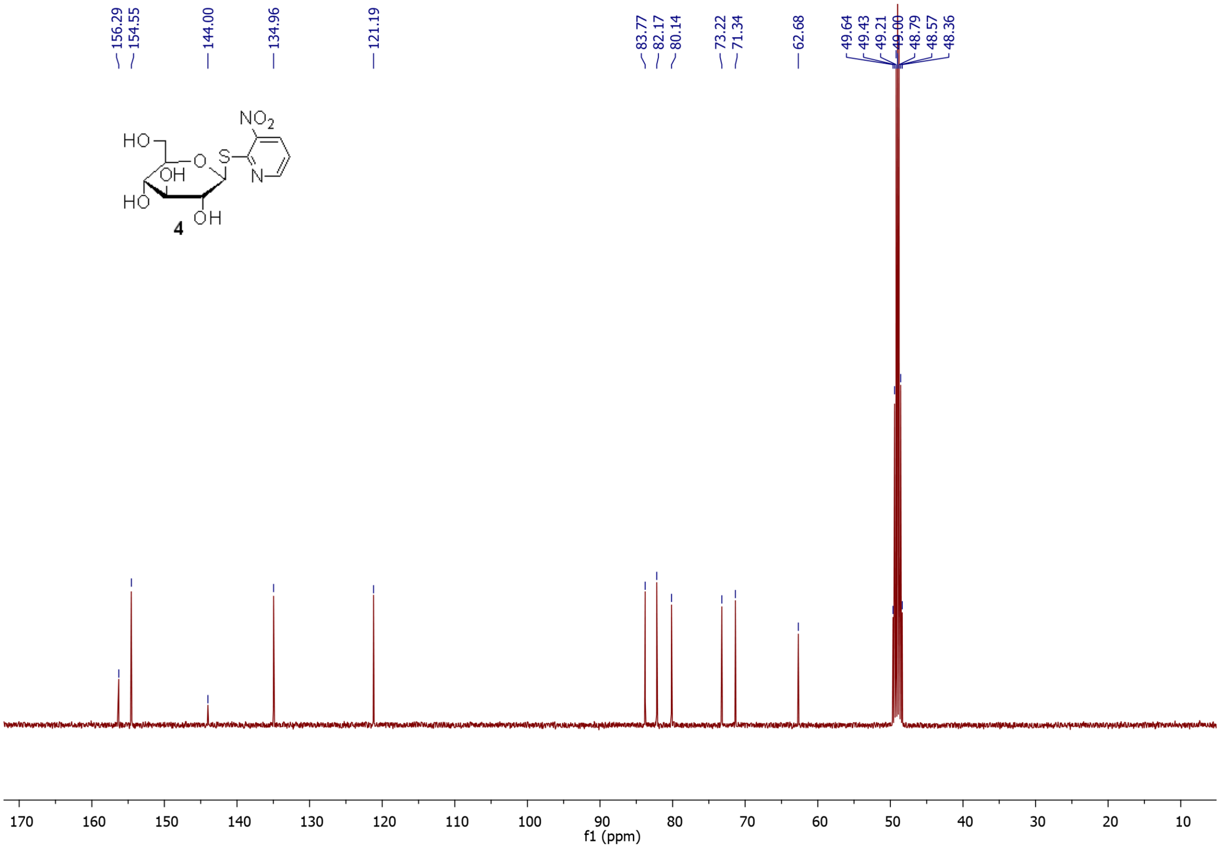

Supplementary Figure 8: ^{13}C NMR spectrum of (3-nitro-2-pyridyl) 1-thio-β-D-glucopyranoside 4.
